# Utility of immunoglobulin isotypes against LID-1 and NDO-LID for, particularly IgG1, confirming the diagnosis of multibacillary leprosy

**DOI:** 10.1590/0074-02760170467

**Published:** 2018-02-26

**Authors:** Pedro Henrique Ferreira Marçal, Lúcia Alves de Oliveira Fraga, Ana Márcia Menezes de Mattos, Laura Menegati, Angélica da Conceição Oliveira Coelho, Roberta Olmo Pinheiro, Euzenir Nunes Sarno, Malcolm S Duthie, Henrique Couto Teixeira

**Affiliations:** 1 Universidade Federal de Juiz de Fora Universidade Federal de Juiz de Fora Instituto de Ciências Biológicas Departamento de Parasitologia, Microbiologia e Imunologia Juiz de ForaMG Brasil Universidade Federal de Juiz de Fora, Instituto de Ciências Biológicas, Departamento de Parasitologia, Microbiologia e Imunologia, Juiz de Fora, MG, Brasil; 2 Universidade Federal de Juiz de Fora Universidade Federal de Juiz de Fora Instituto de Ciências da Vida Governador ValadaresMG Brasil Universidade Federal de Juiz de Fora, Instituto de Ciências da Vida, Governador Valadares, MG, Brasil; 3 Universidade Federal de Juiz de Fora Universidade Federal de Juiz de Fora Faculdade de Enfermagem Departamento de Enfermagem Básica Juiz de ForaMG Brasil Universidade Federal de Juiz de Fora, Faculdade de Enfermagem, Departamento de Enfermagem Básica, Juiz de Fora, MG, Brasil; 4 Fundação Oswaldo Cruz Fundação Oswaldo Cruz-Fiocruz Instituto Oswaldo Cruz Laboratório de Hanseníase Rio de JaneiroRJ Brasil Fundação Oswaldo Cruz-Fiocruz, Instituto Oswaldo Cruz, Laboratório de Hanseníase, Rio de Janeiro, RJ, Brasil; 5 Infectious Disease Research Institute SeattleWA USA Infectious Disease Research Institute, Seattle, WA, USA

**Keywords:** leprosy, serodiagnosis, *M. leprae* antigen, LID-1, natural octyl disaccharide, IgG1 immunoglobulin subclass

## Abstract

**BACKGROUND:**

Leprosy remains a health problem in many countries, with difficulties in diagnosis resulting in delayed treatment and more severe disabilities. Antibodies against several *Mycobacterium leprae* antigens have, however, shown value as diagnostic and/or prognostic markers.

**OBJECTIVES:**

The objective of this study was to evaluate serum immunoglobulin (Ig) IgM and IgG subclass reactivity against three *M. leprae* specific antigens: NDO-HSA, a conjugate formed by natural octyl disaccharide bound to human serum albumin; LID-1, the fusion protein product of the ml0405 and ml2331 genes; and NDO-LID, a combination of LID-1 and NDO.

**METHODS:**

Sera from healthy controls, paucibacillary (PB) and multibacillary (MB) leprosy patients, and their respective household contacts, were evaluated for the presence of antigen-specific IgM, IgG, and IgG subclass antibodies by enzyme-linked immunosorbent assay (ELISA). The sensitivity and specificity of each ELISA were evaluated by receiver operating characteristic (ROC) curve analysis.

**FINDINGS:**

Our data confirm that serum IgM antibodies against NDO-HSA and IgG antibodies against LID-1, as well as IgG/M antibodies against NDO-LID, are markedly increased in MB patients. For the first time, our data reveal a selective increase in IgG1 and IgG3 antibodies against LID-1 and NDO-LID in MB patients, demonstrating that these antibody isotypes are suitable for differentiation between MB and PB patients. ROC curve analysis indicates an improved capacity for diagnosing MB leprosy patients using the detection of IgG antibodies, particularly the IgG1 isotype, specific to LID-1 and NDO-LID over the performance levels attained with NDO-HSA.

**CONCLUSIONS:**

Our findings indicate that serological tests based on the detection of antigen-specific IgG1 antibodies are a useful tool to differentiate MB from PB patients, and indicate the enhanced performance of the LID-1 and NDO-LID antigens in the serodiagnosis of leprosy.

Leprosy is a chronic granulomatous disease that affects the skin, peripheral nerves and nasal mucosa. It is caused by infection with *Mycobacterium leprae*, and has a wide range of clinical and pathological manifestations dictated by the patient’s immune response (Mizoguti et al. 2015). The cellular immune response to *M. leprae* is preserved in the tuberculoid pole (TT), such that patients present a strong production of interferon gamma (IFN-g), reduced levels of specific antibodies, few skin lesions, and low or absent bacillary index. In contrast, weak or absent cellular responses, high levels of antibodies, multiple skin lesions and high bacillary load are observed in the lepromatous (LL) pole. Borderline leprosy forms are present between these two polar extremes and represent a continuous clinical and histopathological range (Ridley & Jopling 1966). For operational purposes, the World Health Organization (WHO) proposed a simplified classification system based on the counting of cutaneous lesions: patients with up to five lesions are considered paucibacillary (PB) and patients with more than five lesions are considered multibacillary (MB). Patients are then prescribed different multidrug therapy (MDT) regimens consisting of daily treatment with supervised doses of rifampicin, clofazimine and dapsone for six to nine months for PB or for twelve to eighteen months for MB (MS/SVS 2016).

Currently the diagnosis of leprosy is achieved essentially by clinical evaluation. Tests such as skin smears and histopathological analyses to directly observe acid-fast bacilli, and the intradermal test measuring delayed type-hypersensitivity responses against dead bacilli, may also contribute to diagnosis. These supplementary tests do not, however, have high sensitivity and high specificity and are also limited by their availability (Contin et al. 2011). To improve leprosy control, it is necessary to develop and integrate simple, sensitive and specific tests that could accelerate diagnosis and assist in classifying patients for treatment. Tests that detect IgM antibodies against phenolic glycolipid-I (PGL-I), or its mimetics di- and trisaccharide analogs NDO and NTP, represent the most advanced tools currently used (Fabri et al. 2015). Several groups are making progress with additional diagnostic markers, including Leprosy Infectious Disease Research Institute (IDRI) Diagnostic-1 (LID-1), a chimeric protein representing the fusion of the genes *ml0405* and *ml2331* (Duthie et al. 2007), and NDO-LID, a conjugate of natural disaccharide octyl (NDO) and LID-1 (Duthie et al. 2014).

Tests that use antigenic targets to quantify specific antibodies can be used as a surrogate marker for bacterial load in leprosy. Although tests detecting particular classes of antibodies may potentially enable a broader assessment of the immune response during infection and provide a diagnostic alternative, serological tests based on the detection of refined IgG subclasses (i.e. IgG1, IgG2, IgG3 and IgG4) against mycobacterial-specific antigens have not been thoroughly explored. While IgM is the first antibody produced in a humoral response, immunoglobulin class switching is a maturation event involving gene rearrangement to generate IgG responses, which is regulated by B cell activators in the presence of T cell-derived cytokines. Immunoglobulin class switching enables antibodies to refine their effector function, thereby contributing to the diversity of the immune response (Tangye et al. 2002, 2013). Importantly, the particular immunoglobulin subclass that emerges can be used as a proxy indicator of the involvement of distinct T helper cell subsets. In humans, interleukin (IL)-4 and IL-13 stimulate the secretion of IgG4 and IgE; IL-10 and IL-21 enhance switching to IgG1 and IgG3; and IFN-g favors IgG3 with suppression of IgG1 (Tangye et al. 2002).

In the present study, sera from PB and MB patients, their respective household contacts, and healthy control individuals, were tested for the presence of antigen-specific IgM and IgG against NDO-HSA, LID-1 and NDO-LID. The sensitivity and the specificity of each particular enzyme-linked immunosorbent assay (ELISA) was evaluated by receiver operating characteristic (ROC) curve analysis. We also conducted a more refined analysis based on detecting the particular IgG subclasses involved in the antigen-specific reactivity.

## SUBJECTS AND METHODS

*Study population -* Patients with MB (n = 18) and PB (n = 20) leprosy were diagnosed at the outpatient unit of the Oswaldo Cruz Foundation in Rio de Janeiro (FIOCRUZ-RJ, Brazil). Leprosy patients were diagnosed by clinical examination according to established dermatological and neurological criteria, with laboratory support. Patients were characterised as PB when presenting five or less skin lesions and negative bacilloscopy, or MB when presenting with more than five lesions and/or positive bacilloscopy, as described by the operational classification adopted by the World Health Organization (MS/SVS 2016). Patients were further characterised according to the Ridley-Jopling classification system of clinical manifestations (Ridley & Jopling 1966). Forty-eight household contacts (HHC) who resided with MB (HHC-MB, n = 28) or PB (HHC-PB, n = 20) leprosy patients, were selected and thoroughly examined for signs of leprosy by physicians with specific training. Twenty healthy individuals from Rio de Janeiro without prior history of mycobacterial disease were included as endemic controls (EC) after undergoing dermatoneurological examinations (Table I).

**TABLE I:** Characteristics of the study participants

Groups	n	Gender (M/F)	Age/years (mean)
Paucibacillary patients (PB)	20	7/13	23-80 (55,7)
Multibacillary patients (MB)	18	11/7	13-74 (45,5)
Paucibacillary household contacts (HHC - PB)	20	9/11	19-68 (38,4)
Multibacillary household contacts (HHC - MB)	28	10/18	15-62 (37,1)
Endemic controls (EC)	20	3/17	20-49 (26,4)

Total	106	40/66	13-80 (40,6)

*Detection of antigen-specific antibodies by ELISA -* Polystyrene 96-well microplates were coated overnight with NDO-HSA, LID-1 and NDO-LID antigens (2 µg/mL) diluted in 0.06 M carbonate buffer (pH 9.6) solution (100 µL per well). After blocking with phosphate-buffered saline (PBS) containing 0.05% Tween 20 (PBS-T) and 1% bovine serum albumin (BSA) for 1 h, wells were washed with PBS-T and serum samples diluted 1:20 in PBS-T containing 10% BSA were added in duplicates (100 µL per well). After incubation at 37ºC for 1 h, plates were washed with PBS-T and then goat anti-human IgG (1:1000) (Invitrogen Corporation, Carlsbad, CA, USA) or mouse anti-human IgG1 (1:1000), anti-IgG2 (1:2000), anti-IgG3 (1:2000) and anti-IgG4 (1:2000) (Southern Biotechnology Associates, Inc., Birmingham, AL, USA) conjugated with horseradish peroxidase (HRP) were added. After 1 h of incubation at 37ºC, plates were washed with PBS-T and then the substrate solution containing 0.5 mg/mL of ortho-phenylenediamine in sodium citrate buffer (0.03% H_2_O_2_, pH 5), was added. The reaction was stopped with 2N H_2_SO_4_ and the optical density measured at 492 nm (Spectramax-190, Molecular Devices, Sunnyvale, CA, USA). The results were expressed by ELISA index (EI), calculated by the formula EI = S/(B + 3 SD), where S is the average optical density value of the duplicate test samples and B corresponds to the average optical density value of the duplicate negative controls plus three times the standard deviation (SD) (Fabri et al. 2015).

*Statistical analysis* - Normal distribution of data was assessed using the Shapiro-Wilk test. Data were then compared using Kruskal-Wallis and *post hoc* Dunn tests. The ROC curve was used to analyse the accuracy values: area under the ROC curve, sensitivity, specificity and likelihood ratios, which were obtained using MedCalc Statistical (Version 5.00.020, Brussels, Belgium). A p < 0.05 was considered statistically significant.

*Ethics* - This study was approved by the Ethical Committee of the Oswaldo Cruz Institute (protocol: 1.896.348). All participants were informed about the study aims and the procedures involved, and were included only after signing an Informed Consent Form in accordance with Resolution 196/1996 of the National Health Council.

## RESULTS

**Fig. 1:**
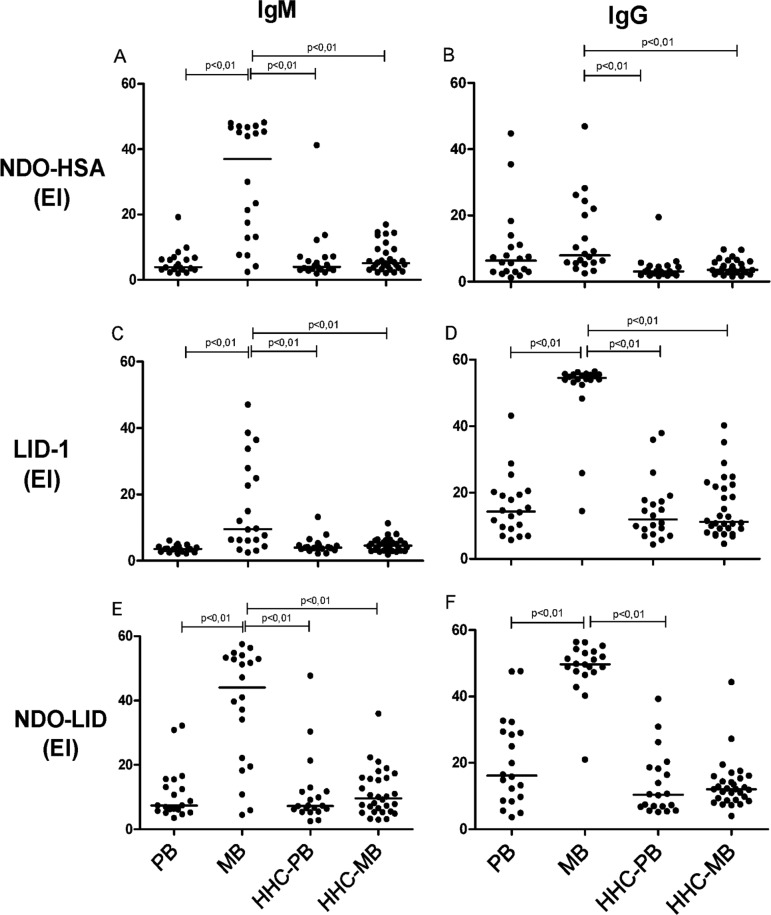
: levels of IgM and IgG against NDO-HSA, LID-1 and NDO-LID in paucibacilary (PB) and multibacilary (MB) leprosy patients and their household contacts (HHC-PB and HHC-MB). Each point represents the result obtained from an individual serum sample, with the bars representing the median. EI = Elisa index.

*Detection of IgM and IgG antibodies against M. leprae antigens* - Given that NDO-HSA, LID-1 and NDO-LID represent glycolipid, protein, and glycolipid/protein antigen targets, respectively, we first assessed the presence of either IgM or IgG antibodies capable of reacting to these *M. leprae* antigens among the various cohorts. Antibodies in the serum from clinically confirmed MB and PB leprosy patients, and in the serum from the contacts of both MB and PB patients (representing individuals with higher exposure risk but lacking overt clinical symptoms; HHC-MB and HHC-PB, respectively), were evaluated. As expected, the levels of IgM and IgG serum antibodies against NDO-HSA, LID-1 and NDO-LID were markedly increased in MB patients, but not PB patients, in comparison with either HHC-MB or HHC-PB (Fig. 1). As expected, IgM antibodies had higher reactivity with NDO, whereas IgG antibodies had robust reactivity against LID-1. A small subset of MB patients demonstrated IgM antibodies that were reactive with LID-1. High levels of both IgM and IgG antibodies against NDO-LID, a conjugate combining both NDO and LID-1, were detected in the majority of MB patients (Fig. 1). No significant antigen-specific responses were observed in samples from the HHC-MB and HHC-PB groups, regardless of whether IgM or IgG antibodies were being evaluated [Fig. 1,

Supplementary data(Figure)].

**Fig. 2:**
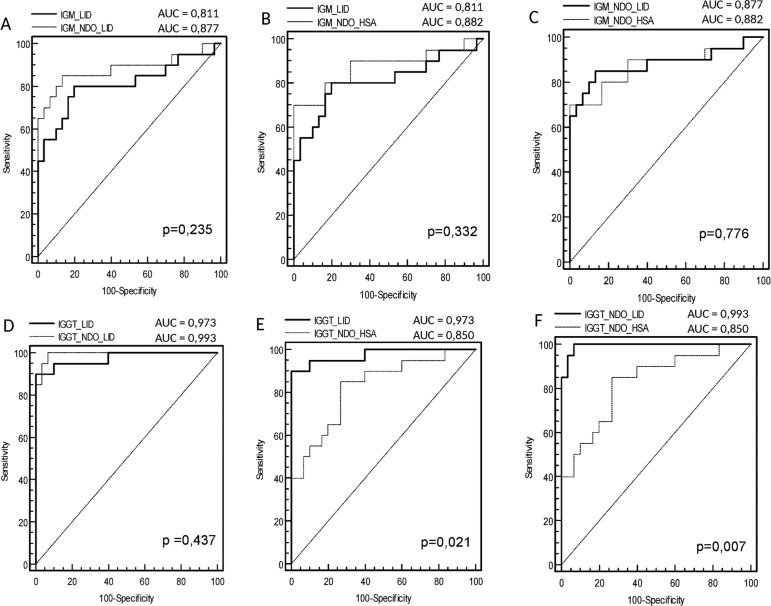
: receiver operating characteristic (ROC) curves for IgM and IgG responses to LID-1 versus NDO-LID (A, D), LID-1 versus NDO-HSA (B, E) and NDO-LID versus NDO-HSA (C, F).

ROC curve analysis of data from the MB group indicated that the area under the curve (AUC) for IgM was slightly, but not significantly, higher for NDO-HSA [AUC = 0.882] than for either LID-1 [AUC = 0.811] or NDO-LID [AUC = 0.877] (Fig. 2). With respect to IgG responses, the AUC against either LID-1 [AUC = 0.973] or NDO-LID [AUC = 0.993] were both significantly higher than that observed for NDO-HSA [AUC = 0.850]. The ROC curve analysis showed that at the optimal cut off (i.e. the point located nearest to the left upper corner of the ROC curve Cartesian space) NDO-HSA-specific IgM levels provided a sensitivity of 70% with a specificity of 98%, yielding a positive likelihood ratio of 35 and a negative likelihood ratio of 0.31. Similar analyses indicated that NDO-HSA detection with IgG yielded 85% sensitivity and 78% specificity, rates lower than those achieved with LID-1 (90% sensitivity and 100% specificity) and NDO-LID (95% sensitivity and 98% specificity). In terms of the likelihood ratio generated when IgG was detected by ELISA, the positive and negative values for NDO-HSA were 3.86 and 0.19; for LID-1 were 48.0 and 0.10; and for NDO-LID were 47.5 and 0.5, respectively (Table II). These results indicate an improved potential for diagnosing MB leprosy patients using the detection of IgG antibodies specific to LID-1 and NDO-LID over those achieved with NDO-HSA.

**TABLE II:** Sensitivity and specificity of the evaluated antigens in the serodiagnosis of multibacillary leprosy

Antigens	Isotypes	Cutoff^*a*^	Sensitivity^*b*^	Specificity^*b*^	+LR^*c*^	-LR^*c*^
NDO-HSA	IgM	16.93	70	98	35.0	0.31
	IgG	5.25	85	78	3.86	0.19
	IgG1	1.31	75	88	6.25	0.28
	IgG2	1.56	65	78	2.95	0.45
	IgG3	1.82	70	84	4.37	0.36
LID-1	IgM	6.06	80	82	4.44	0.24
	IgG	40.26	90	100	48.0	0.10
	IgG1	18.10	90	100	11.0	0.10
	IgG2	2.49	80	88	6.67	0.23
	IgG3	6.13	85	74	3.27	0.20
NDO-LID	IgM	18.02	85	86	6.07	0.17
	IgG	39.28	95	98	47.5	0.05
	IgG1	5.15	90	90	9.00	0.11
	IgG2	2.36	85	82	4.72	0.18
	IgG3	5.14	90	88	7.50	0.11

*a*: cutoff, sensitivity and specificity data were determined based on the analysis of receiver operating characteristic (ROC) curves; *b*: the values of sensitivity and specificity were determined according to the point of the ROC curve nearest to the point of sensitivity and specificity equal to 100%; *c*: +LR and -LR = positive and negative likelihood ratio (LR).

**Fig. 3:**
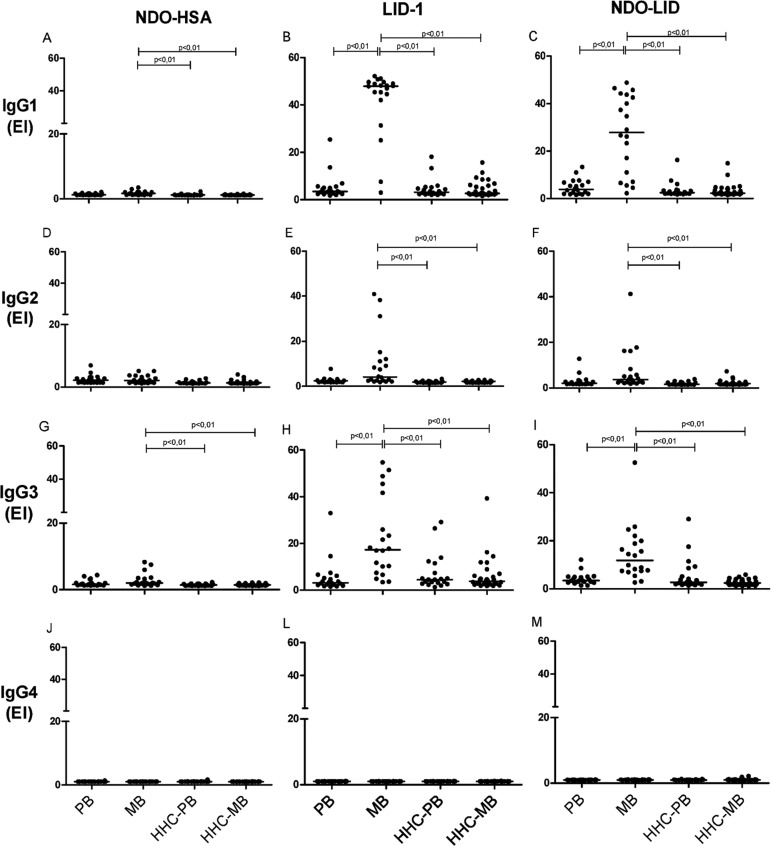
: levels of IgG1, IgG2, IgG3 and IgG4 against NDO-HSA, LID-1 and NDO-LID in paucibacillary (PB) and multibacillary (MB) leprosy patients and their household contacts of PB (HHC-PB) and MB (HHC-MB). Each point represents the result obtained from an individual serum sample, with the bars representing the median. EI = Elisa index.

*Differential presence of IgG subclasses against M. leprae antigens* - Given the important role of adaptive T helper cells in determining the manifestation of leprosy, we next evaluated the IgG isotype composition of the antigen-specific serum antibody responses. In sera from MB patients, antigen-specific IgG1 antibodies were the most readily detected, followed by IgG3 then IgG2, while antigen-specific IgG4 responses were undetectable (Fig. 3). Levels of IgG1 and IgG3 against LID-1 and NDO-LID were significantly higher in the sera of MB patients when compared with sera of PB patients. Furthermore, the dichotomous immune responses of MB and PB patients were not apparent upon analyses of antigen-specific IgG subclasses, because none of the IgG subclasses examined were particularly prevalent in the sera of PB patients. ROC curve analysis revealed high IgG1 reactivity to LID-1 and NDO-LID in MB patients, with sensitivity of 90% for both antigens, and specificities of 100 and 90%, respectively (Table II), reinforcing their potential in confirming the diagnosis of MB leprosy.

## DISCUSSION

Diagnostic tools to help in the early detection of leprosy and provision of adequate treatment are essential to interrupt the progression of the disease towards physical disabilities (Goulart et al. 2008). Currently, the laboratory diagnosis of leprosy is technically difficult, raising the need for both rapid and sensitive techniques that can detect all clinical forms and for tests that can be applied in the field (Geluk et al. 2011, Bahmanyar et al. 2016). Our findings confirm that serum IgM and IgG antibodies against NDO-HSA and LID-1, as well as the NDO-LID that combines these antigens, are markedly increased in MB patients, while our more detailed analyses indicate that the antigen-specific IgG response comprises predominantly IgG1 and IgG3 subclasses.

The detection of antibodies against PGL-I (or its mimetic NDO) and LID-1 has shown to be useful as diagnostic and prognostic indicators of leprosy (Duthie et al. 2014, Freitas et al. 2015). As expected, IgM antibodies had higher reactivity to NDO-HSA, whereas IgG antibodies showed robust reactivity against the LID-1 antigen. It is well known that IgM responses to protein antigens are relatively low, whereas non-protein antigens such as NDO preferentially stimulate an IgM response (Buhrer-Sekula et al. 2009, Kumar et al. 2014). ROC curve analysis, which is used to assess the performance of a diagnostic test, indicated a sensitivity of 70% and a specificity of 98% for IgM against NDO-HSA in MB patients. It is also well known that MB patients present with high bacillary loads and elevated antibody responses (Sampaio et al. 2011, Kumar et al. 2014). The increased humoral response in leprosy patients, however, is not able to eliminate *M. leprae*, thus favoring disease progression and bacillary spread. In the present work, the ROC curve analysis showed that the IgG and IgG1 tests against LID-1 and NDO-LID antigens were very accurate in discriminating MB leprosy patients from their household contacts, showing higher sensitivity and specificity. Duthie et al. (2013), using one rapid quantitative serological test, observed that NDO-LID can assist in the diagnosis and monitoring of MB leprosy, detecting a significant number of patients in the earlier stages of disease development. A study conducted in Southwest China provided evidence of the effectiveness of detecting anti-LID-1 responses as an early diagnostic tool for MB leprosy in household contacts of leprosy patients (Qiong-Hua et al. 2013). Serology studies from different areas in Brazil confirmed anti-LID-1 tests as tools for the detection of MB leprosy and for the identification of individuals with subclinical *M. leprae* infection (Hungria et al. 2012, Fabri et al. 2015).

It is documented that HHCs are at higher risk of *M. leprae* infection and development of disease than the general population, and thus a greater positivity to *M. leprae* antigens is expected among HHCs compared with that in the general population (Carvalho et al. 2015, Penna et al. 2016). The anti-PGL-I seropositivity in HHCs indicated a six-times higher risk of developing leprosy (Goulart et al. 2008). Our analyses found no significant differences among the seroreactivities in the HHC-MB, HHC-PB, and EC groups [

Supplementary data(Figure)] with regard to IgM or IgG antibody levels against the particular antigens studied. Similarly, when evaluating the sensitivity of the NDO-LID test, Cardoso et al. (2013) did not detect differences between HHC-PB and HHC-MB cohorts. Curiously, in our evaluations the EC group presented higher total IgG levels against LID-1 and NDO-LID than the HHC groups [

Supplementary data(Figure)]. In agreement with these findings, another study observed a higher rate of anti-NDO-HSA and anti-NDO-LID positivity in the general population than in HHCs, as well as a positive correlation between anti-NDO-HSA, anti-LID-1, and anti-NDO-LID antibodies in HHCs and in the general population (Fabri et al. 2015). The higher endemicity rate in the region from which the EC individual samples were collected probably contributed to this higher seropositivity rate (Moet et al. 2008).

Studies of IgG subclasses in leprosy patients are relatively scarce. For the first time, IgG subclass responses against NDO-HSA, LID-1 and NDO-LID were investigated. Among the four subclasses of IgG, a selective increase in IgG1 and IgG3 against LID-1 and NDO-LID antigens was detected in MB patients (IgG1 > IgG3 > IgG2 > IgG4), demonstrating that these antibody isotypes are more suitable for differentiation between MB and PB patients. IgG subclass reactivity to NDO-HSA, in contrast, was very low in all groups, with levels of IgG1 and IgG3 against NDO-HSA only slightly increased in MB patients. In accordance with our findings, elevated IgG1 and IgG3 responses to an *M. leprae* 18K recombinant antigen were previously detected across the leprosy spectrum, and were not associated with a polyclonal IgG activation. Among the four subclasses of IgG, IgG1 and IgG3 are biologically the most active and may serve as biomarkers of disease progression in leprosy (Hussain et al. 1994, 1995), 1995). Similar to our findings with the LID-1 and NDO-LID antigens, very low IgG4 reactivity to *M. leprae* 18K recombinant antigen was described. Human IgG4 and IgE can bind to mast cells and have been shown to be associated with Th2 activation and disease progression with intracellular pathogens. The lower IgG4 and IgE responses against *M. leprae* antigens contradicts the hypothesis that Th2 bias occurs in leprosy (Hussain et al. 1994), an issue that deserves further investigation.

Cytokines signals can drive class switch recombination events to produce distinct antibody classes. In mice IgG2a is considered a functional equivalent of human IgG1, and both have been shown to be regulated by IFN-g, a Th1-secreted cytokine (Finkelman et al. 1988). However, augmentation of IgG1 antibodies against the ML10k antigen was observed in lepromatous patients in the absence of a detectable Th1 response, suggesting that in this case the switch to IgG1 might be derived from alternative cell sources (Hussain et al. 1999). Th2 and Th17-like T follicular helper (T_FH_) subsets were shown to produce large levels of IL-21, a cytokine involved in CD8 T-cell priming in tuberculosis that induces switching predominantly to IgG1 and IgG3 isotypes (Tangye et al. 2013, Booty et al. 2016). Moreover, class switching to IgG1 in humans has also been shown to be dependent on IL-10 (Briere et al. 1994, Malisan et al. 1996), a cytokine secreted by macrophages and Th2 cells. The roles of IL-21 and IL-10 in IgG class switching in MB leprosy patients remain to be determined.

As well as detecting individuals already exhibiting overt symptoms of leprosy, an ideal diagnostic test would detect individuals who are subclinically infected with *M. leprae* but are likely to develop the clinical manifestations of the disease (Bahmanyar et al. 2016). Since serum antigen-specific antibody responses can closely reflect *M. leprae* infection levels, detection of antibodies against LID-1 and NDO-LID may represent a simpler and less invasive technique with which to estimate bacterial burden than intrusive skin slit smears. These antibody-based tests could thereby contribute to accurate leprosy diagnosis, especially in areas where histopathological exams are not available. In conclusion, our results indicate that the detection of IgG, and particularly IgG1, reactivity to LID-1 and NDO-LID may be useful in defining the clinical forms of leprosy.
