# Supplementary material for: Utility of immunoglobulin isotypes against LID-1 and NDO-LID for, particularly IgG1, confirming the diagnosis of multibacillary leprosy
Source: Mem Inst Oswaldo Cruz. 2018 Feb 26;113(5):e170467. doi: 10.1590/0074-02760170467 (PMC5851060; doi:10.1590/0074-02760170467)
Supplement: Supplementary file 1 [file 0074-0276-mioc-113-5-e170467-suppl01.pdf]

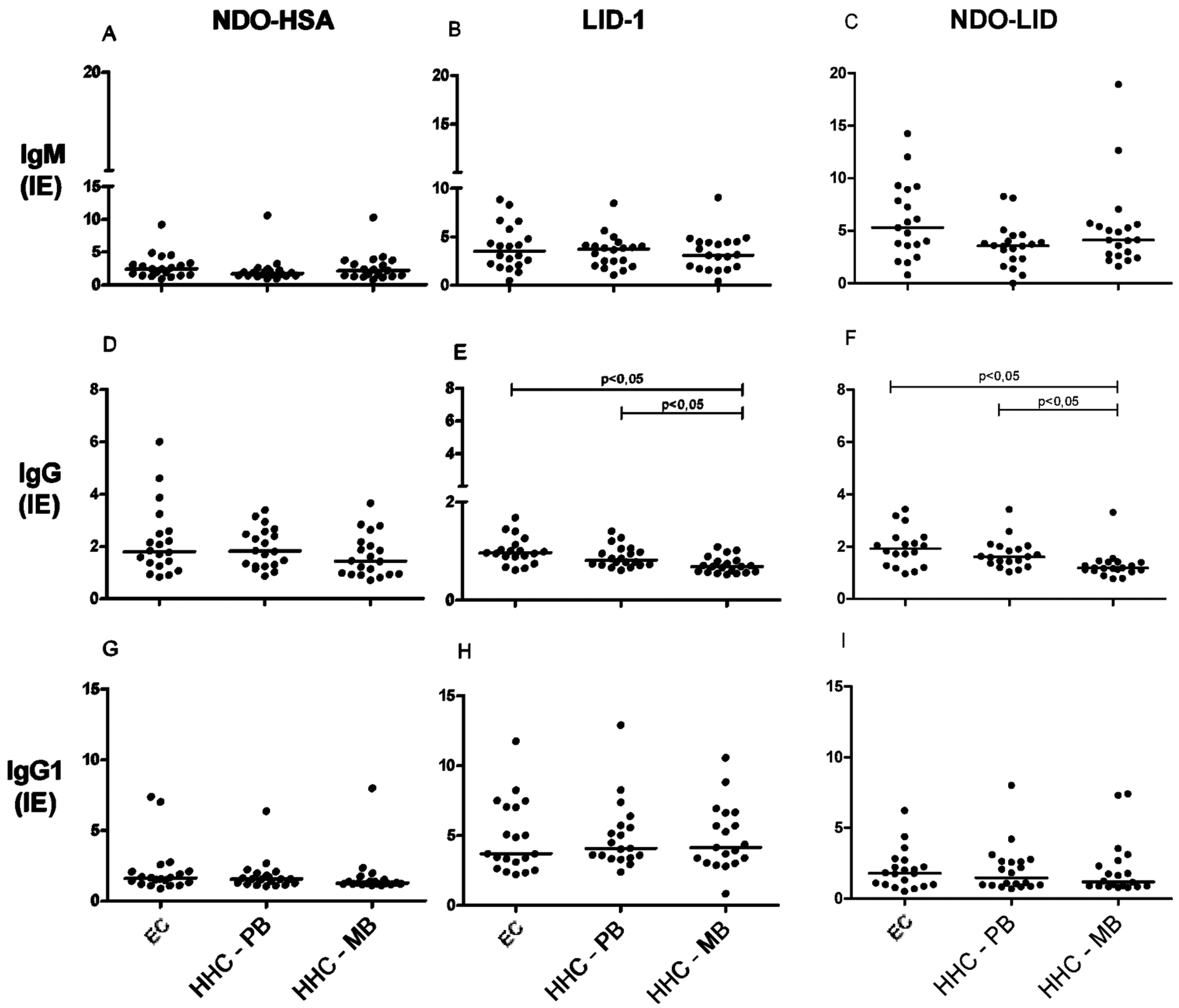

Levels of IgM, IgG and IgG1 against NDO-HSA, LID-1 and NDO-LID in endemic controls (EC) and household contacts of paucibacillary (HHC-PB) and multibacillary (HHC-MB) leprosy. Each point represents the result obtained from an individual serum sample, with the bars representing the median. EI = Elisa index.

CC BY
